# Minimally invasive pedicle screw fixation combined with percutaneous vertebroplasty for preventing secondary fracture after vertebroplasty

**DOI:** 10.1186/s13018-015-0172-1

**Published:** 2015-03-07

**Authors:** Yu-Tong Gu, Dong-Hui Zhu, Hai-Fei Liu, Feng Zhang, Robert McGuire

**Affiliations:** Department of Orthopaedics, Zhongshan Hospital of Fudan University, Shanghai, 200032 China; Department of Orthopaedics, Shanghai Electric Power Hospital, Shanghai, 200050 China; Department of Orthopaedics, The Affiliated Hospital of Medical College, Qingdao University, Qingdao, Shandong China; Division of Plastic Surgery, University of Mississippi Medical Center, Jackson, Mississippi USA; Department of Orthopaedics, University of Mississippi Medical Center, Jackson, Mississippi USA

**Keywords:** Osteoporosis, Thoracolumbar vertebral fracture, Vertebroplasty, Percutaneous surgery, Pedicle screw fixation, Minimally invasive spinal surgery

## Abstract

**Background:**

Percutaneous vertebroplasty (PVP) and percutaneous kyphoplasty (PKP) could give rise to excellent outcomes and significant improvements in pain, analgesic requirements, function, cost, and incidence of serious complications for thoracolumbar osteoporotic vertebral compression fractures (VCFs). But some studies showed the recurrent fracture of a previously operated vertebra or adjacent vertebral fracture after PVP or PKP. The purpose of this study was to compare minimally invasive pedicle screw fixation (MIPS) and PVP with PVP to evaluate its feasibility and safety for treating acute thoracolumbar osteoporotic VCF and preventing the secondary VCF after PVP.

**Methods:**

Sixty-eight patients with a mean age of 74.5 years (ranging 65 ~ 87 years), who sustained thoracic or lumbar fresh osteoporotic VCFs without neurologic deficits underwent the procedure of PVP (group 1, *n* = 37) or MIPS combined with PVP (group 2, *n* = 31). Visual analog scale pain scores (VAS) were recorded and Cobb angles, central and anterior vertebral body height were measured on the lateral radiographs before surgery and immediately, 1 month, 2 months, 3 months, 6 months, 1 year, and 2 years after surgery.

**Results:**

The patients were followed for an average of 27 months (ranging 24–32 months). The VAS significantly decreased after surgery in both groups (*P* < 0.005). The central and anterior vertebral body height significantly increased (*P* < 0.005), and the Cobb angle significantly decreased (*P* < 0.05) immediately after surgery in both groups. No significant changes in both the Cobb angle correction and the vertebral body height gains obtained were observed at the end of the follow-up period in group 2. But the Cobb angle significantly increased (*P* < 0.005), and the central and anterior vertebral body height significantly decreased (*P* < 0.005) 2 years after surgery compared with those immediately after surgery in group 1, and there were five patients with new fracture of operated vertebrae and nine cases with fracture of adjacent vertebrae.

**Conclusions:**

MIPS combined with PVP is a good choice for the treatment of acute thoracolumbar osteoporotic VCF, which can prevent secondary VCF after PVP.

## Introduction

Osteoporosis and its associated fractures have become an important health issue because of an aging population. Osteoporotic vertebral compression fractures (VCFs) can cause debilitating pain and functional decline necessitating prolonged bed rest and high-dose narcotics. Percutaneous vertebroplasty (PVP), minimal invasive injection of bone cement into the fractured vertebral body, can stabilize osteoporotic VCFs with resultant relief of associated local back pain. Percutaneous kyphoplasty (PKP) is a modification of PVP [1], in which a inflatable instrument is inserted into the vertebral body through the pedicle to restore the height of a collapsed vertebral body and create a cavity inside before the cement is injected. A lot of studies have shown that PVP and PKP could give rise to excellent outcomes and significant improvements in pain, analgesic requirements, function, cost, and incidence of serious complications [2-8], although Kallmes et al. [9] and Buchbinder et al. [10] reported that improvements in pain and pain-related disability associated with osteoporotic VCFs in patients treated with PVP were similar to the improvements in a control group with a sham procedure.

However, secondary VCFs after PVP or PKP have been reported including further compression of previously operated vertebrae [11-15] and newly developed fractures in adjacent vertebrae [3,16-23] with no additional trauma. Lavelle and Cheney [12] found a 10% incidence rate for recurrent fracture of the operated vertebra after PKP. Kim and Rhyu showed that the incidence of recompression in treated vertebrae was 12.5% [15]. Jensen et al. reported that the percentage of new adjacent vertebral fracture occurrence after PVP is 20%–25% [16,17]. Kim et al. [22] found that 51.9% of 114 patients who underwent PVP subsequently suffered from adjacent vertebral fractures. Rho et al. reported that 27 (18.4%) in 147 patients treated with PVP or PKP had subsequent symptomatic new VCFs and 66.7% of the 27 patients had a new VCF on the adjacent vertebra [23].

There are a few contributing factors to secondary VCF after PVP or PKP such as age, bone mineral density (BMD), body mass index (BMI), preoperative osteonecrosis, intervertebral cleft (IVC), pre-existing fracture, treatment modality, amounts of cement injected, restoration rate of vertebral height, non-PMMA-endplate-contact (NPEC), and intradiscal cement leakage, but these remain speculative [15,16,22,23]. The effective strategy avoiding secondary VCF after PVP or PKP has not yet been found. Clinical studies have shown that combined PKP and pedicle screw osteosynthesis to treat thoracic and lumbar burst fractures could achieved maintenance of sagittal curve and vertebral height correction in the injured vertebrae [24-26]. In this study, we designed a technique of minimally invasive pedicle screw fixation (MIPS) combined with PVP [27] for treatment of acute thoracolumbar osteoporotic VCF to prevent the occurrence of secondary VCF after PVP. This method was compared with PVP to evaluate its feasibility and safety.

## Methods

The clinical study proposal was approved by Zhongshan Hospital Ethical Committee (the medical ethical committee of the authors’ hospital). From November 2010 to August 2011, 73 patients with an osteoporotic VCFs (AO classification A-1 of the thoracic or lumbar spine without neurologic deficits were selected for this study in our hospital. Preoperative clinical assessments, neurological tests, and pain assessments using the visual analog scale (VAS) were obtained. The radiological tests performed prior to surgery included standard anteroposterior and lateral roentgenograms of the fractured vertebrae, CT scans with axial, sagittal and coronal reconstruction, and magnetic resonance imaging (MRI) for checking that the spinal cord and the posterior ligamentous complex were intact. All patients had the presence of one recent (<7 days) thoracolumbar osteoporotic VCF, defined as more than 15°of local kyphosis and/or 25% of vertebral height loss, and edema, a fracture line, or both within the vertebral body on MRI. The exclusionary criteria were the presence of more than two vertebral fracture, spinal cancer, neurological signs, spinal cord compromise, discal damage on MRI, medical conditions that would make the patient ineligible for emergency decompressive surgery if needed, previous vertebroplasty, inability to give informed consent, and a likelihood of noncompliance with follow-up.

The patients were randomly assigned to two groups: group 1, treated with PVP; group 2, treated with MIPS combined with PVP. Patient demographic, including age, gender, BMI, and BMD, was obtained. The mean BMI is calculated as the weight in kilograms divided by the square of the height in meters (kg/m^2^); the lumbar spine BMD (T-score) was measured by dual-energy X-ray absorptiometry.

The patients were operated on a priority rather than an emergency basis within a week after trauma. In group 1, PVP (Ruibang, Shanghai, China) was performed under local or general anesthesia. Minimally invasive pedicle screw-and-rod reduction and fixation (EXPEDIUM, Depuy Spine, Raynham, Massachusetts, USA and XIA, Stryker Spine, Bordeaux, Cestas, France) and PVP (Ruibang, Shanghai, China) were performed under general anesthesia in group 2. Antibiotic prophylaxis (2 g of cefazoline during surgery and 2 g two times in the following 24 h) was used. Patients were positioned in the prone position on a radiolucent operating table with surgical bolsters placed under the thorax and iliac crests in order to induce spinal lordosis and facilitate the reduction of the fracture. The involved vertebrae were identified, and the skin was marked under lateral fluroscopic control before beginning the surgical procedure.

## Surgical procedure

### Group 1

**Figure 1:**
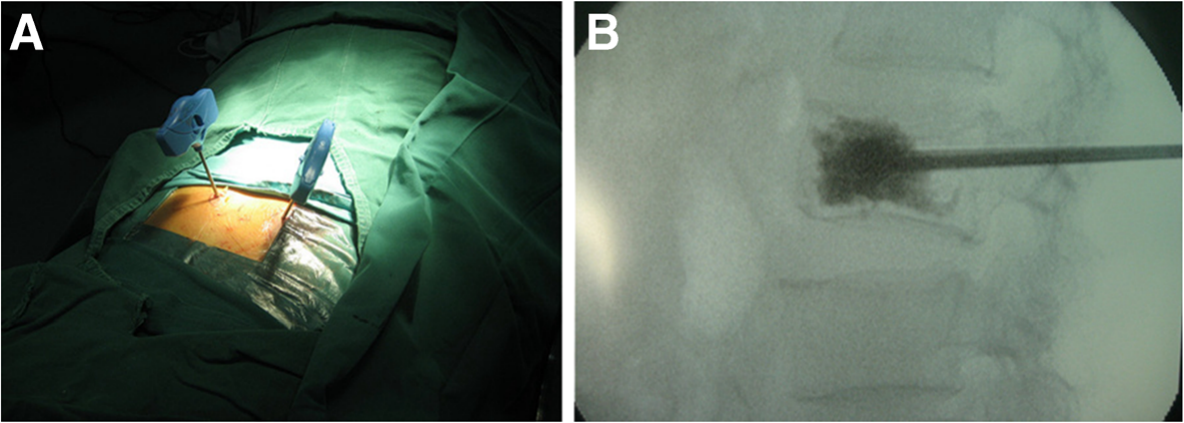
**PVP for fractured vertebral body. (A)** Insertion of 13-gauge needles into the fractured vertebral body through the pedicles under fluoroscopic guidance. **(B)** Injection of bone cement into the target vertebral body under constant fluoroscopy.

Thirteen gauge needles were passed into the anterior central aspect of the fractured vertebral body through the pedicles under fluoroscopic guidance (Figure 1A). For the PVP procedure, bone cement (polymethyl methacrylate (PMMA)) was injected under constant fluoroscopy into the target vertebral body through the previously placed needles until the cement approaches the posterior aspect of the vertebral body or leaks into an extraosseous space, such as the intervertebral disc or an epidural or paravertebral vein (Figure 1B).

### Group 2

**Figure 2:**
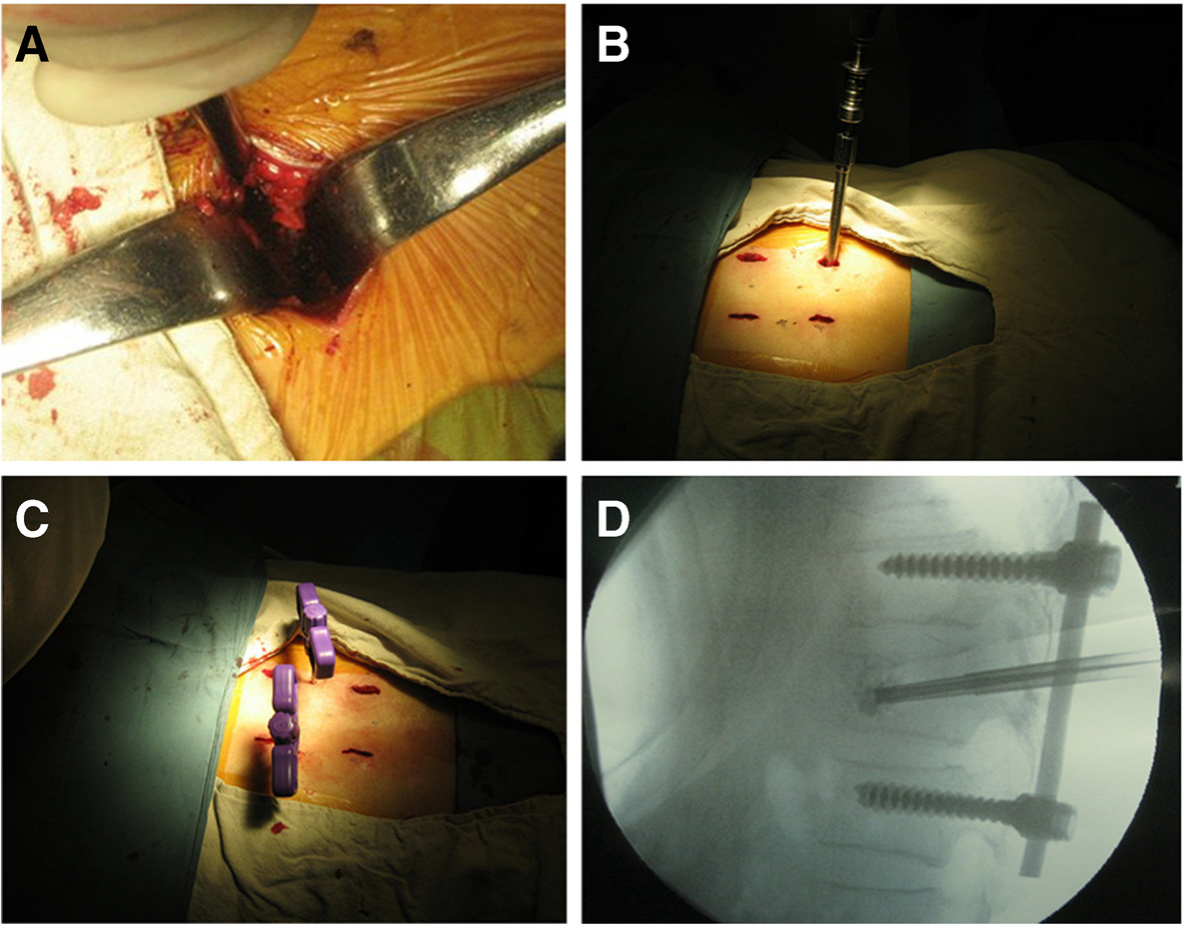
**Minimally invasive pedicle screws fixation and PVP for** f**ractured vertebral body. (A)** Exposion of superior articular facet and root of transverse process through the minimal-access in a paraspinal sacrospinalis muscle-splitting (Wiltse) approach. **(B)** Placement of pedicle screw into the adjacent vertebrae to fractured one with minimally invasive technique. **(C)** Insertion of 13-gauge needles into the fractured vertebral body under fluoroscopic guidance. **(D)** Injection of cement after minimally invasive pedicle screw-and-rod reduction and fixation.

Non-cannulated pedicle screws were placed into the adjacent vertebrae to fractured one with minimally invasive technique. The minimal access in a paraspinal sacrospinalis muscle-splitting (Wiltse) approach [28] was performed to expose superior articular facet and root of transverse process (Figure 2A). The entry site to the pedicle was located at the junction between the lateral border of the superior articular facet and the bisecting midline of the transverse process. Once the pedicle has been identified, either a pedicle probe or a handheld curette was used to enter the pedicle. Preoperative anteroposterior and lateral roentgenograms and CT scans through the pedicles of the vertebral body to be instrumented are studied to determine the correct angle of entry in both the coronal and sagittal planes. The pedicle integrity was verified in all four quadrants to be sure that a solid tube of bone exists and that violation into the spinal canal or inferiorly into the neuroforamen has not occurred. Four pedicle screws of appropriate length are then introduced into the vertebral body via the pedicle to engage at least 75% of the vertebral body anteriorposterior width (Figure 2B). Anteroposterior and lateral x-rays are taken to confirm their position and the 13-gauge needles are then passed into the anterior central aspect of the fractured vertebral body through the pedicles under fluoroscopic guidance (Figure 2C). Two rods of the appropriate size were placed over the pedicle screws through subcutaneous soft tissues and muscles. The fracture was reduced by the combination of the method of installation and distraction applied between two screws as necessary. And then the PVP procedure was undertaken to inject bone cement into the involved vertebral body (Figure 2D).

No external braces were prescribed after the operation. The patients were mobilized as soon as feasible after surgery. After leaving hospital, patients were encouraged to resume their daily routine and followed-up as outpatients at the hospital ward.

## Clinical and radiographic evaluation

All the patients underwent clinical assessments to check for neurological deficits and VAS pain assessments immediately, 1 month, 2 months, 3 months, 6 months, 1 year, and 2 years after surgery. Anteroposterior and lateral radiographs (first supine then standing later) were obtained to evaluate the reduction of the fracture, the distribution of the cement, and the position of the implants. CT scan was performed to check that no cement leakage had occurred into the spinal canal immediately after operation. Cobb angles and central and anterior vertebral body height were measured on the lateral radiographs. The fractured and restored heights were calculated as a percentage of the estimated, intact vertebral body height by averaging the respective central and anterior heights from the adjacent levels [24]. The radiographic measurements of pre- and post-operation were performed by the same doctor.

## Statistical analysis

Independent data, including age, BMI, BMD, and injected cement quantity, were compared between groups 1 and 2 using the Mann-Whitney *U* test. Differences in sex ratios and fracture level ratios between two groups were compared using the chi-square test. Independent-samples *t* test was used to compare VAS, central and anterior vertebral body height, and Cobb angle between two groups. Comparison of pre- and postoperative measurements was performed using one-way analysis of variance for independent samples followed by Turkey post hoc analysis for multiple comparison procedures. Statistically significant differences were defined at a 95% confidence level. The values are given as mean ± standard deviation. The SPSS software (SPSS Inc., Chicago, IL, USA) supported statistical evaluation.

## Results

Group 1 is comprised of 37 patients treated with PVP. Group 2 included 31 patients who underwent MIPS combined with PVP. Table 1 summarizes the comparison of clinical data between the two groups. There was no significant difference in age, gender, BMI, BMD, or fracture level between groups 1 and 2. The VAS, central and anterior vertebral body height, and Cobb angle before surgery showed no significant difference between the two groups. (Tables 2, 3, 4, and 5).

**Table 1:** **Comparison of clinical data between groups 1 and 2**

		**Group 1**	**Group 2**	***P*** **value**
Age (years)		75.1 ± 5.5	73.9 ± 6.4	0.355
Gender (F/M)		24/13	23/8	0.572
BMI (kg/m^2^)		23.1 ± 3.3	23.3 ± 3.4	0.883
BMD (T-score)		−3.4 ± 0.8	−3.5 ± 0.9	0.517
Fracture level	T_11_	3	2	0.597
	T_12_	9	12	
	L_1_	19	14	
	L_2_	6	3	
PMMA amount (ml)		5.7 ± 1.1	6.1 ± 1.4	0.232
duration of operation (minutes)		43.4 ± 5.0	74.7 ± 8.6	0.000^*^
blood loss (ml)		5.5 ± 1.5	70.2 ± 4.7	0.000^*^
stay at hospital (days)		3.2 ± 0.4	5.3 ± 1.0	0.000^*^
Follow-up period (months)		27.4 ± 2.5	27.2 ± 2.5	0.742

^*^ *P* < 0.05.

**Table 2:** **VAS pain assessments of two groups**

**Group**	**Preoperative**	**Postoperative**	**1 month**	**2 months**	**3 months**	**6 months**	**1 year**	**2 years**
1	9.1 ± 1.1	2.2 ± 1.4	2.5 ± 1.2	2.2 ± 1.2	1.7 ± 1.0	1.3 ± 0.9	0.9 ± 1.1	0.9 ± 1.1
2	9.1 ± 1.0	2.4 ± 0.9	1.7 ± 0.8	1.2 ± 0.6	1.0 ± 0.7	0.9 ± 0.7	0.6 ± 0.6	0.5 ± 0.6

Values are expressed as the mean ± SD. There was significant difference (*P* < 0.005) 1 month, 2 months, and 3 months after surgery between the two groups. The VAS after surgery was significantly lower (*P* < 0.005) than that of before surgery in two groups. The VAS immediately after surgery was significantly higher (*P* < 0.05) than that of 6 months, 1 year, and 2 years in group 1. The VAS immediately after surgery was significantly higher (*P* < 0.05) than that of 1 month, 2 months, 3 months, 6 months, 1 year, and 2 years in group 2.

**Table 3:** **Central vertebral body height of two groups (%)**

**Group**	**Preoperative**	**Postoperative**	**1 month**	**2 months**	**3 months**	**6 months**	**1 year**	**2 years**
1	43.5 ± 7.6	66.1 ± 7.1	56.4 ± 6.8	56.1 ± 6.9	56.1 ± 6.9	56.1 ± 6.9	56.1 ± 6.9	56.1 ± 6.9
2	43.4 ± 7.4	72.8 ± 6.5	70.6 ± 6.3	69.5 ± 6.7	69.3 ± 6.7	69.3 ± 6.7	69.3 ± 6.7	69.3 ± 6.7

Values are expressed as the mean ± SD. There was significant difference (*P* < 0.005) after surgery between the two groups. The central height after surgery was significantly higher (*P* < 0.005) than that of before surgery in two groups. The central height immediately after surgery was significantly higher (*P* < 0.005) than that of 1 month, 2 months, 3 months, 6 months, 1 year, and 2 years in group 1.

**Table 4:** **Anterior vertebral body height of two groups (%)**

**Group**	**Preoperative**	**Postoperative**	**1 month**	**2 months**	**3 months**	**6 months**	**1 year**	**2 years**
1	49.8 ± 8.1	74.7 ± 7.0	63.6 ± 6.7	63.4 ± 6.7	63.4 ± 6.6	63.4 ± 6.6	63.4 ± 6.6	63.4 ± 6.6
2	49.7 ± 8.0	81.2 ± 6.6	79.7 ± 6.6	78.1 ± 6.6	77.9 ± 6.6	77.8 ± 6.5	77.8 ± 6.5	77.8 ± 6.5

Values are expressed as the mean ± SD. There was significant difference (*P* < 0.005) after surgery between the two groups. The anterior height after surgery was significantly higher (*P* < 0.005) than that of before surgery in two groups. The anterior height immediately after surgery was significantly higher (*P* < 0.005) than that of 1 month, 2 months, 3 months, 6 months, 1 year, and 2 years in group 1.

**Table 5:** **Local kyphosis of two groups (°)**

**Group**	**Preoperative**	**Postoperative**	**1 month**	**2 months**	**3 months**	**6 months**	**1 year**	**2 years**
1	18.1 ± 3.9	11.3 ± 3.8	14.8 ± 3.9	15.1 ± 3.8	15.1 ± 3.9	15.1 ± 3.9	15.1 ± 3.9	15.1 ± 3.9
2	18.2 ± 3.9	7.3 ± 3.2	7.9 ± 3.2	7.9 ± 3.2	8.0 ± 3.2	8.0 ± 3.2	8.0 ± 3.2	8.0 ± 3.2

Values are expressed as the mean ± SD. There was significant difference (*P* < 0.005) after surgery between the two groups. The Cobb angle after surgery was significantly less than that before surgery in group 1 (*P* < 0.05) and group 2 (*P* < 0.005). The Cobb angle immediately after surgery was significantly less (*P* < 0.005) than that of 1 month, 2 months, 3 months, 6 months, 1 year, and 2 years in group 1.

None of the patients were found to have any postoperative neurological complications. The amount of cement injected was 5.7 ± 1.1 ml in group 1 and 6.1 ± 1.4 ml in group 2 (*P* = 0.232). The duration of operation was 43.4 ± 5.0 min in group 1 and 74.7 ± 8.6 min in group 2 (*P* = 0.000). There was blood loss of 5.5 ± 1.5 ml in group 1 and 70.2 ± 4.7 ml in group 2 (*P* = 0.000). The stay at hospital was 3.2 ± 0.4 day in group 1 and 5.3 ± 1.0 day in group 2 (*P* = 0.000). The patients were followed for 27.4 ± 2.5 months in group 1 and for 27.2 ± 2.5 months in group 2 (*P* = 0.742).

The VAS significantly decreased after surgery in both groups (*P* < 0.005) and VAS of group 2 was significantly lower than that of group of PVP 1 month, 2 months, and 3 months after surgery (*P* < 0.005). The VAS of group 1 was higher than that of group of PVP 6 months, 1 year, and 2 years after surgery, although there was no significant difference between the two groups (Table 2).

**Figure 3:**
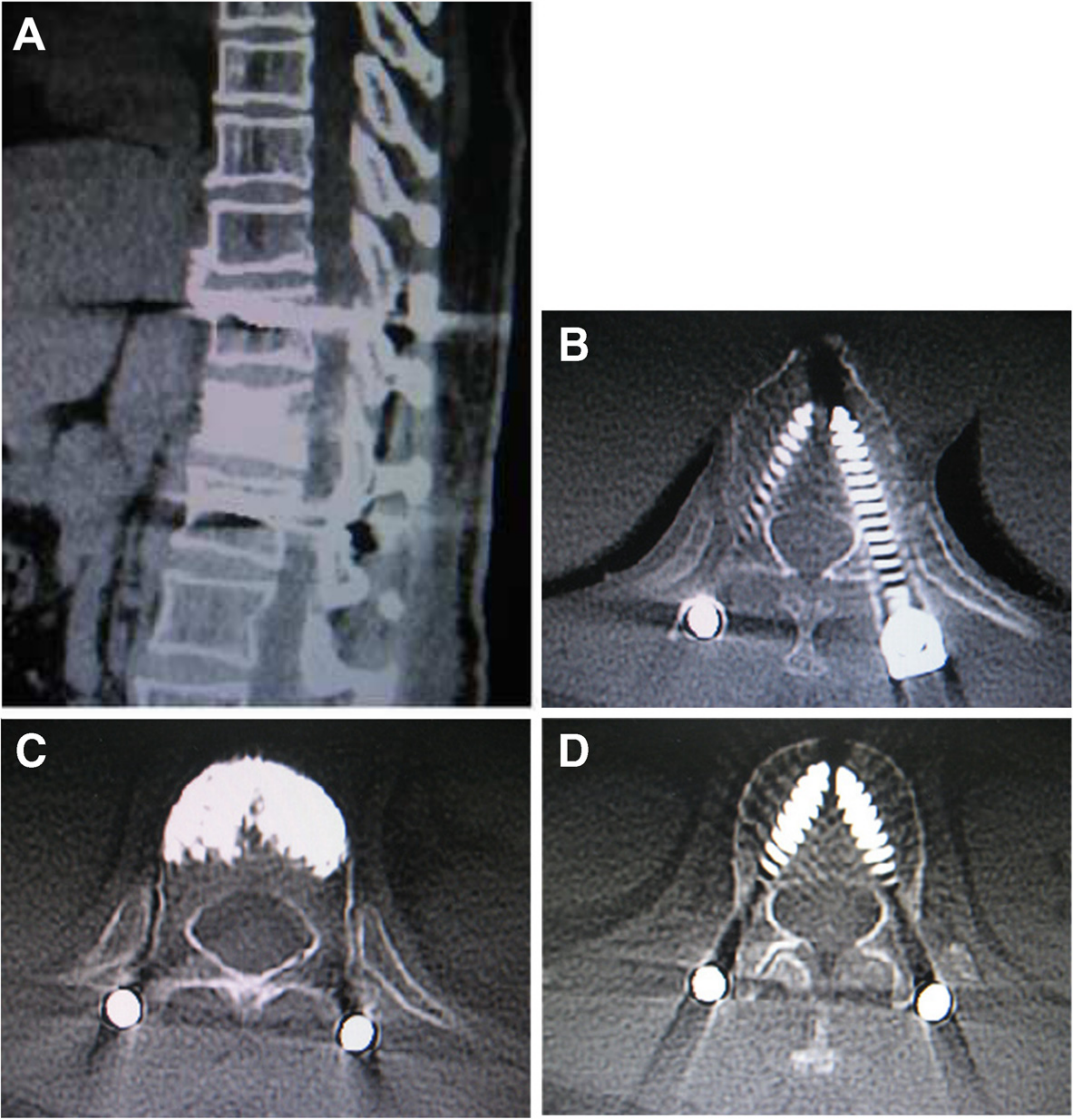
**Postoperative CT scanographic images.** Sagittal **(A)** and axial CT-scan **(B)**, **(C)**, **(D)** immediate postoperative reconstruction, verification of pedicle screws positioning and search for cement leakage.

In all patients, the postoperative radiographs and scanographic images demonstrated a good position of the pedicle screw construct and the cement in the fractured vertebral body (see examples in Figure 3). The CT scan images also showed that no cement leakage had occurred into the spinal canal. Four cases of anterior or lateral leakage in group 1, and two cases of lateral leakage in group 2 were diagnosed without clinical consequences. On postoperative examinations, no signs of significant cement resorption or bridging of intervertebral segment were noticed.

**Figure 4:**
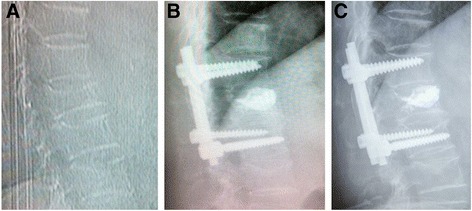
**Female patient of 62 years with T12 VCF undergoing minimally invasive pedicle screws fixation and PVP.** Preoperative lateral view of the fracture **(A)**, postoperative lateral view **(B)**, and evolution after 2-year follow-up **(C)** without significant loss of correction.

**Figure 5:**
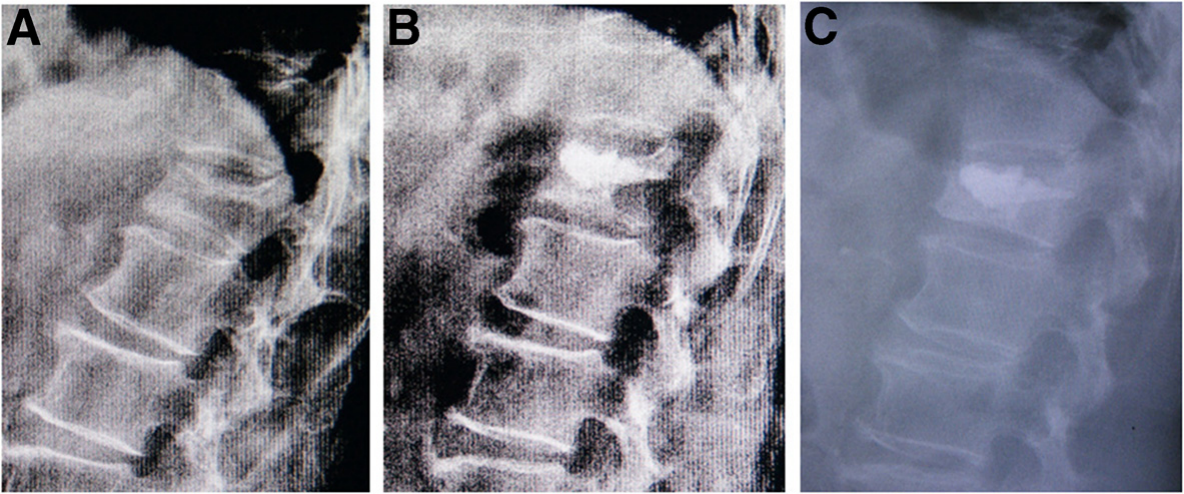
**Female patient of 67 years with L1 VCF undergoing PVP.** Preoperative lateral view of the fracture **(A)**, postoperative lateral view **(B)**, and reoccurrence fracture of operated vertebra after 1-month follow-up **(C)**.

The central and anterior vertebral body height significantly increased (*P* < 0.005), and the Cobb angle significantly decreased (*P* < 0.05) immediately after surgery in both groups. There were significant differences (*P* < 0.005) after surgery between groups 1 and 2. No significant changes in both the Cobb angle correction and the vertebral body height gains obtained were observed at the end of the follow-up period in group 2 (Figure 4). But the Cobb angle significantly increased (*P* < 0.005), and the central and anterior vertebral body height significantly decreased (*P* < 0.005) 2 years after surgery compared with those immediately after surgery in group 1. The results of all the statistical tests carried out are given in Tables 3, 4, and 5. There were five patients with new fracture of operated vertebrae (Figure 5) and nine cases with fracture of adjacent vertebrae in group 1 and no patients with secondary fracture in group 2. No hardware failure was seen in any patient following the instrumentation and PVP. The refractured patients with more back pain underwent conservative treatment such as bed rest and medication.

## Discussion

Osteoporotic VCFs usually lead to back pain, loss of height, kyphotic deformity, and a reduction in quality of life [29]. PVP and PKP are cement augmentation procedures used to control pain and restore function in patients with osteoporotic VCFs that are refractory to conservative treatment [1-8,30]. But some studies showed the recurrent fracture of a previously operated vertebra or adjacent vertebral fracture after PVP or PKP [3,11-23].

Fuentes et al. [26] used PKP associated with percutaneous short-segment cannulated pedicle screw osteosynthesis in 18 patients of burst vertebral fractures without neurological deficits. The mean vertebral height was improved by 25%, and a mean improvement of 11.28°in the local kyphotic angle was obtained. No significant changes in the results obtained were observed at the end of the follow-up period. Verlaan et al. [24,25] performed balloon kyphoplasty in combination with pedicle screw instrumentation to treat thoracic and lumbar burst fractures. The postoperative radiographs and computer tomography or magnetic resonance images demonstrated a good fracture reduction and filling of the bone defect without unwarranted bone displacement. There was no instrumentation failure or measurable loss of sagittal curve and vertebral height correction in the follow-up. We designed the MIPS combined with PVP technique [27] for osteoporotic VCF in order to prevent the occurrence of secondary VCF after PVP.

The feasibility and relative safety of MIPS combined with PVP were confirmed by the fact that postoperative radiographs and scanographic images showed that the screws and cement were all properly positioned in the patients of group 2. None of the patients were found to have any postoperative neurological complications. Like all surgical interventions, pedicle screw stabilization is not devoid of risks, since it can cause nerve injuries. The pedicle must be carefully probed in all four quadrants to be sure that a solid tube of bone exists and that violation into the spinal canal or inferiorly into the neuroforamen has not occurred before the pedicle screws were implanted into the vertebrae with minimally invasive technique under direct vision in our study. Cement injection also involves risks of complications including cement leakage into the spinal canal, which is greater when the posterior wall has been damaged. During the PVP procedure, we injected bone cement into the target vertebral body under constant fluoroscopy, which must be stopped if the cement got close to the posterior aspect of the vertebral body or leaked into an extraosseous space. All of these measures were taken to avoid the occurrence of neurological deficits and guarantee the safety of operation.

MIPS combined with PVP was compared with PVP to evaluate its rate of secondary fracture after PVP in this study. The results showed that 18.2 ± 3.9° of Cobb angle before surgery significantly decreased to 7.3 ± 3.2° immediately after surgery in group 2 (*P* < 0.005). The central vertebral body height significantly increased from 43.4 ± 7.4% before surgery to 72.8 ± 6.5% of the estimated intact central height immediately after surgery (*P* < 0.005). The anterior vertebral body height significantly increased from 49.7 ± 8.0% before surgery to 81.2 ± 6.6% of the estimated intact anterior height immediately after surgery (*P* < 0.005). It is more important that the correction obtained of both the Cobb angle and the vertebral body height was stable in time with a minimal loss of correction at final follow-up (0.7° of kyphosis, 3.5% of central vertebral height, and 3.4% of anterior vertebral height after 2 years) which seemed to occur during the 2 months after surgery. No fracture of the operated or adjacent vertebral body was found in group of MIPS combined with PVP. But the Cobb angle significantly increased (*P* < 0.005), and the central and anterior vertebral body height significantly decreased (*P* < 0.005), 2 years after surgery compared with those immediately after surgery in group of PVP. There were five (13.5%) patients with new fracture of operated vertebrae and nine (24.3%) cases with fracture of adjacent vertebrae, which is similar to other studies [4,11-23]. Although there was no significant difference in VAS 6 months, 1 year, and 2 years after surgery between groups, VAS in group of PVP was higher than those in group of MIPS and PVP. These scores included high VAS of refractured patients with more back pain, who underwent conservative treatment such as bed rest and medication.

The fracture was reduced by the combination of the method of installation and proper distraction applied between two screws as necessary before PVP in group 2, which is better than only by installation supported by the results that the Cobb angle, the central and anterior height of group 2 was significantly better (*P* < 0.005) than those of group 1 immediately after surgery. Short-segment pedicle screw instrumentation is a well described technique to reduce and stabilize thoracic and lumbar spine fractures [31,32]. It is a relatively easy procedure but the means of augmenting the anterior column are limited. Hardware failure and a loss of reduction are recognized complications caused by insufficient anterior column support [33-35], even in young patients in whom resistance to pedicle screw pull-out is high. It is known that cement-based vertebroplasty can restore, even increase, strength and stiffness after VCFs in osteoporotic specimens [36-40]. Vertebroplasty with cement after posterior instrumentation might reduce the load on the pedicle screw, hardware failure, and anterior column collapse [41]. This conclusion was also supported by the results of our study in which there is no hardware failure in any patient during follow-up after instrumentation insertion and PVP, although the mean age of these patients was 73.9 years. These data gave us more confidence to use the pedicle screw fixation in elderly patients.

**Figure 6:**
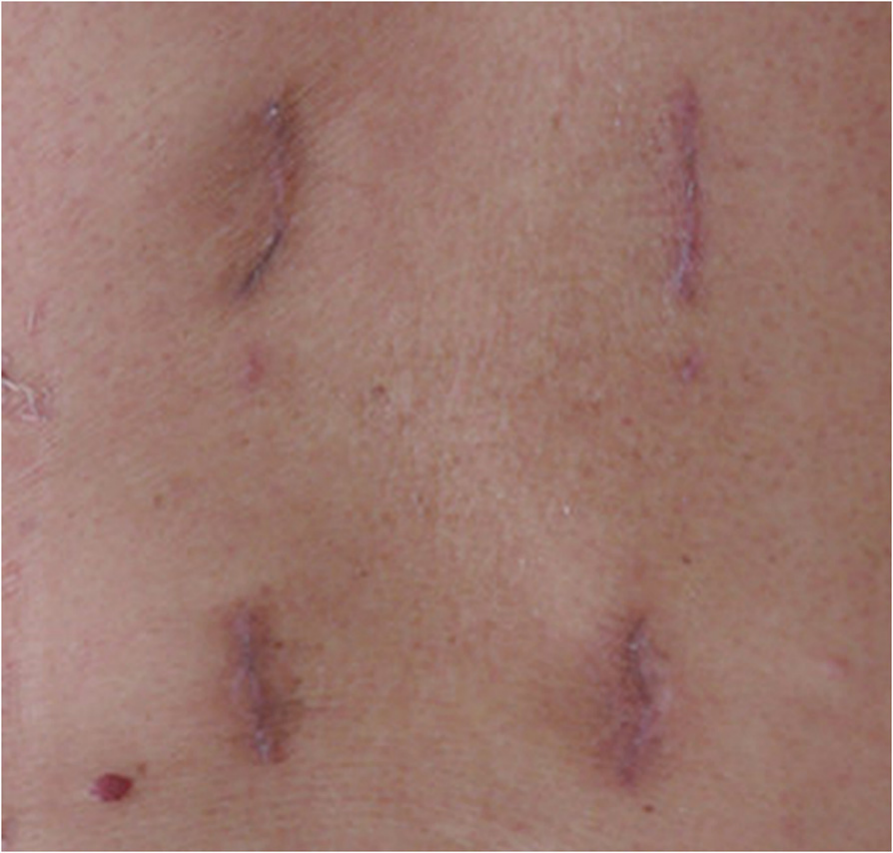
**Minimally invasive access: cosmetic results obtained after the insertion of pedicle screws and PVP (1 month after surgery).**

In this series, the minimal-access in the paraspinal sacrospinalis muscle-splitting (Wiltse) approach [28] was performed to insert non-cannulated pedicle screws into the vertebrae and two rods of the appropriate size were placed over the pedicle screws through subcutaneous soft tissues and muscles. Unlike the traditional midline incision, Wiltse approach protected the attachment of muscle to bone, avoid disruption of the supraspinous and interspinous ligaments, provided a more direct approach to the transverse processes and pedicles, and decreased bleeding and postoperative pain [42-44]. In the group of MIPS and PVP, the duration of the operation was 74.7 ± 8.6 min, the blood loss was 70.2 ± 4.7 ml, and the stay at hospital was 5.3 ± 1.0 days. These values were considered acceptable although there were significant differences compared with the group of PVP (*P* = 0.000). The pain intensity level on the VAS significantly dropped from 9.1 ± 1.0 of pre-operation to 2.4 ± 0.9 (*P* < 0.005) immediately after the operation in the group of MIPS and PVP, which was similar to that in the group of PVP. The results show that MIPS only devote the limited additional trauma to PVP (Figure 6). If the patient with refracture after PVP has severe back pain or neurological compression symptom, the additional treatment such as revision surgery will consume more manpower, material, and financial resources, and the patient will suffer more trauma. Compared with percutaneous pedicle screws, minimally invasive non-cannulated pedicle screw fixation has the incisions of similar size, but easier manipulation and less fluoroscopic monitor during the operation. The common pedicle screws used in this technique were much cheaper than the percutaneous cannulated ones.

## Conclusions

MIPS combined with PVP is a good choice for the treatment of acute thoracolumbar osteoporotic VCF, which can prevent the occurrence of secondary VCF after PVP.

## Consent

Written informed consent was obtained from all patients for the publication of this report and any accompanying images.
